# Sodium picosulfate/magnesium citrate versus 4L split-dose polyethylene glycol for bowel cleansing prior to colonoscopy in high fibre diet African patients

**DOI:** 10.11604/pamj.2021.40.43.28389

**Published:** 2021-09-17

**Authors:** Emeka Ray-Offor, Kalanne Ada Opusunju

**Affiliations:** 1 Digestive Disease Unit, Oak Endoscopy Centre, Port Harcourt, Rivers State, Nigeria,; 2 Colorectal and Minimal Access Surgery Unit, Department of Surgery, University of Port Harcourt Teaching Hospital, Port Harcourt, Rivers State, Nigeria

**Keywords:** Colonoscopy, bowel cleansing agents, outcome

## Abstract

**Introduction:**

an adequate bowel preparation is essential for good mucosal inspection during colonoscopy. This study aims to compare the efficacy of two validated oral lavage solutions for colonoscopy preparation in African patients.

**Methods:**

a prospective observational study of patients undergoing colonoscopy in a referral endoscopy facility in Port Harcourt, Nigeria, using sodium picosulfate magnesium citrate (SPMC) and 4L split-dose polyethylene glycol (PEG). Variables collated were sociodemographic, primary indication, comorbidities, Aronchick bowel preparation scale, polyp/adenoma detection, caecal intubation and outcome. Statistical analysis was performed using IBM SPSS version 20.

**Results:**

one hundred and twenty-four patients received PEG prior to colonoscopy and SPMC in 175 patients. The age range was from 22 to 92 years; mean age of 53.8 ± 14.2 years for PEG group and 55.3 ± 13.2 years for SPMC group (p=0.361). There were 215 males and 84 females. An excellent/good bowel preparation scale was recorded in 77 (62%) PEG group and 130 (74.3%) for SPMC group (p=0.592). PEG was predominantly used in the early years of endoscopists practice with the odds ratio (OR) of no polyp detection in the PEG vs SPMC groups as 1.64 (confidence interval CI 1.06-2.55) versus 0.76 (CI 0.62-0.92), respectively (p=0.016). For no adenoma detection, OR was 4.18 (CI 1.12-15.60) versus OR 0.63 (CI 0.52-0.75), respectively (p=0.012).

**Conclusion:**

there is similar efficacy profile using either split volume PEG or SPMC prior to colonoscopy in these African patients. Polyp and adenoma detection rates are highly dependent on the expertise of the endoscopist.

## Introduction

Colonoscopy is a sensitive tool for screening and diagnosis of colorectal cancer with the advantage of possible polyp removal and resection of early-stage cancer. Emerging reports indicate a rising incidence of gastrointestinal cancers, especially colorectal cancer, in sub-Saharan Africa [1, 2]. The quality of bowel preparation is a major determinant of successful outcome of colon study by colonoscopy. Many factors impact on the efficacy of bowel preparation agents including patient compliance with the preparation instructions provided, health literacy and socioeconomic status [3]. Other confounding factors are use of narcotics, chronic constipation, diabetes and timing of bowel preparation in relation to colonoscopy [4]. Suboptimal bowel cleansing can result in small or flat lesions being missed, shortened surveillance times, significant impediment in progression of colonoscope, incomplete study and increased likelihood of complications with more sedatives and analgesics being required [5-7].

Bowel cleansing agents in use are multiple with differing rates of effectiveness, tolerability and side effect. Basically, a good bowel preparation should be safe, palatable and efficacious [8]. Polyethylene glycol electrolyte solution (PEG-ELS), an iso-osmotic laxative, has been in use as an oral lavage agent for bowel preparation prior to colonoscopy for the last four decades [9]. There is a challenge of compliance to 4L full volume for bowel cleansing, so to improve efficacy splitting the dose has become increasingly popular [10]. Further attempts to improve compliance and efficacy include the introduction of adjuncts e.g. bisacodyl [11]. An alternative agent is sodium picosulfate magnesium citrate (PSMC) which is a dual action laxative. This is commonly sold in a composition comprising sodium picosulfate, magnesium oxide and citric acid. When dissolved in water, the magnesium oxide and citric acid combine to form sodium picosulfate (stimulant laxative) and magnesium citrate (osmoticlaxative) [12].

A low-residue diet for the two days preceding colonoscopy is proven to improve bowel cleansing prior to colonoscopy [13]. More so, in Africans with a typical high fibre diet pattern, there is the need for compliance to a minimum of 2-day dietary restriction unlike the one-day dietary restriction advocated by European gastroenterology society [14]. Currently, there is high level evidence from meta-analyses of international studies validating the efficacy and safety of PEG and SPMC for bowel preparation prior to colonoscopy however lacking in African patients with typical high fibre diet pattern [15, 16]. There is a paucity of African literature on bowel preparation for colonoscopy using PEG and SPMC. This study aims to compare the efficacy of two oral lavage bowel preparation agents prior to colonoscopy in Africa´s most populous country-Nigeria.

## Methods

**Study design:** this is a prospective observational study of consecutive patients undergoing colonoscopy at a referral endoscopy facility in Port Harcourt metropolis, Nigeria, from January 2015 to October 2019. The centre receives referrals of patient from within Rivers State and nearby states of the Niger delta region of Nigeria. An ethical approval was obtained from Oak Endoscopy Centre Ethics Review Committee (OEM/2014/A004). Informed consent was obtained from study patients according to Helsinki declaration. The inclusion criteria were consecutive adult patients undergoing bowel preparation with sodium picosulfate magnesium citrate (group A) and 4L split-dose polyethylene (group B) prior to colonoscopy. There was non-ready supply of bowel preparation agents which were imported in batches of thirties for one available preparation agent at a time by the study centre then restocked before expiration of stock. Hence, there was assignment of bowel cleansing agents to patients in groups of 30s based on available stock. In group A, 2-sachet pack of sodium picosulfate magnesium citrate (Picolax, Ferring Pharmaceuticals UK) consisting of sodium picosulfate 0.01 g, magnesium oxide 3.5 g and anhydrous citric acid 12 g, was given to patients at pre-endoscopy consultation. There is an exothermic reaction that occurs when magnesium oxide reacts with anhydrous citric acid to form magnesium citrate [17]:

One sachet content was mixed in a glass of water and taken in the evening prior to procedure and a second on the morning of procedure. The second group of patients received 4L split-dose polyethylene glycol-electrolyte solution PEG-ELS (KleanPrep, Norgine Ltd Oxford, UK; Polyethylene glycol 236 g, sodium sulphate 22.74 g, sodium bicarbonate 6.74 g, sodium chloride 5.86 g and potassium chloride 2.97 g). Two sachets were mixed in 2L of water and administered the evening before procedure and a repeat of this on the morning of procedure. In both groups, an adjunct- bisacodyl 15 mg daily, for two days preceding procedure was administered.

Additionally, a restriction of diet to low residue and a liberal intake of clear fluids by all patients was instructed during preparation. Excluded from the study were patients below the age of 18 years and patients using other bowel cleansing agents including castor oil/bisacodyl, the few cases of 2L PEG+ ascorbic acid and PEG 3350 non-electrolyte solution. An informed consent was obtained for the colonoscopy procedure after clear explanation of procedure and risks to patient with an explanatory leaflet also given to literate patients. Also, follow-up phone calls were made from the study centre in the days preceding procedure to ensure compliance.

**Endoscopy equipment:** the endoscopy equipment used was Karl Storz (Germany) video colonoscope 13925PKS, camera unit, 100W Xenon light source/pump, HD monitor and AIDA data capture device.

**Procedure:** all colonoscopies were performed by the same endoscopist (ER-O). A sedation/analgesia protocol of intravenous benzodiazepine (diazepam 2.5 mg - 10 mg) and an opioid analgesic-(pentazocine 30 mg) were primarily used. General anaesthesia administered by an anaesthesiologist, was offered to patients that desired deep sedation. A digitally guided insertion of endoscope was performed and the colonoscope advanced by upward and downward deflection of the wheel and torque through the rectum with the use of the right/left wheel as needed further on. A gentle air insufflation or water distension was used to dilate collapsed bowel with the primary goal of reaching the caecum. The colon was carefully inspected during the withdrawal. The patients were observed for a minimum of 15 minutes post procedure before discharge.

**Outcome assessment:** the primary outcome studied was quality of colon cleanliness. This quality of bowel cleansing was assessed by the endoscopist using a 4-grade scale of Aronchick scale [18]. Bowel preparation was graded as excellent when little or no liquid residue was seen and no supplementary cleaning or manoeuvres needed with > 95% of mucosa inspected. The cleansing was graded as good when liquid residue requiring moderate suction or change in position for adequate examination and > 90% of mucosa inspected. A fair grade was ascribed when liquid and semisolid residue requiring flushing for adequate examination of colonic mucosa were seen but >90% of mucosa was inspected. Finally, a poor grade was recorded when liquid and semisolid residue requiring flushing for adequate examination of colonic mucosa and < 90% of mucosa was inspected. In all, an adequate bowel preparation was recorded with a grade of excellent or good. The secondary outcomes of interest were tolerability, adverse effect, caecal intubation and polyp/adenoma detection rates. Tolerability was defined as the number of patients who ingested the entire bowel cleaning preparation.

**Statistical analysis:** data analysis was performed using IBM SPSS statistics for Windows, version 20.0. Armonk, NY, USA. The frequency distribution of data was summarized as numbers, mean and percentages as appropriate. The mean age of patients in both groups was compared with independent t test and risk of no polyp or adenoma detection was calculated using Mantel-Haenszel common odds ratio estimate. Categorical variables in both groups were compared using Pearson´s Chi-square test. Statistical significance was set at P<0.05.

## Results

A total of 299 patients were included in study. One hundred and twenty-four patients received 4L split-volume PEG-ELS prior to colonoscopy and 175 patients received SPMC. The age of patients ranged from 22 to 92 years; mean age of 53.8 ± 14.2 years for PEG-ELS group and 55.3 ± 13.2 years for SPMC group. There were 215 (71.9%) males and 84 (28.1%) females (Table 1). The male to female ratio in patients who received PEG was 2.4: 1 and 2.7: 1 in the SPMC group. Statistically, there was no difference in the age and sex distribution between the 2 groups (p = 0.361 and p = 0.632 respectively).

**Table 1:** sociodemographic of study population

Variables	PEG-ELS (n=124)	SPMC (n=175)	Total
**Age**			
<20 years	0	0	0
20 - 29 years	3	4	7
30 - 39 years	15	18	33
40 - 49 years	28	29	57
50 - 59 years	35	70	105
60 - 69 years	29	27	56
≥70 years	14	27	41
**Sex**			
Male	87	128	215
Female	37	47	84
**Educational status**			
Primary	1	5	6
Secondary	8	10	18
Tertiary	53	106	159
Not stated	62	54	116
**Comorbidity**			
Diabetes mellitus	2	8	11
Hypertension	35	56	91
Major psychiatric illness	0	0	0

**Figure 1:**
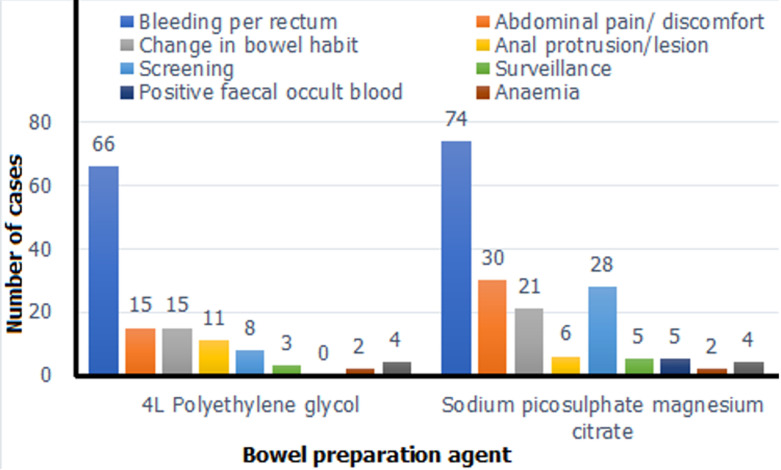
primary indication for colonoscopy

Overall, patients with secondary and post-secondary education were 179 (60.0%). In the PEG group, 10 (8.1%) patients were recorded with comorbidity of diabetes mellitus and 43 (34.7%) as hypertensives: predominantly on calcium channel blockers (amlodipine). In contrast, among patients that received SPMC, 70 (40.0%) hypertensives and 23 (13.1%) were diabetics (Table 1). The most common indication for colonoscopy was bleeding per rectum (Figure 1).

Tolerability in both patient groups was excellent as only 2 patients in PEG group failed to complete full dose of bowel preparation agents and only one failed completion of dose in the SPMC group (98.4% vs 99.4% respectively). An excellent/good bowel preparation scale was recorded in 77 (62.1%) patients for PEG-ELS and 130 (74.3%) patients for SPMC (p=0.592). Caecal intubation rates of 84.7% and 86.9% were recorded for PEG and SPMC group of patients, respectively. However, there was statistical difference in polyp/adenoma detection rates (p=0.014 and p=0.004 respectively) (Table 2). An odds ratio (OR) of no polyp detection in the PEG vs SPMC groups of 1.64 (confidence interval CI 1.06-2.55) and 0.76 (CI 0.62-0.92) respectively (p=0.016). For no adenoma detection in the PEG vs SPMC group of patients OR was 4.18 (CI 1.12-15.60) and OR 0.63 (CI 0.52-0.75), respectively (p=0.012).

**Table 2:** bivariate analysis of patient groups

Variables	PEG-ELS (n=124)	SPMC (n=175)	P value
Mean age (years)	54.1 ± 13.5	55.3 ± 13.2	0.465
**Sex**			
Male	87	128	0.572
Female	37	47	
Tolerability	122 (98.4%)	174 (99.4%)	0.373
**Bowel preparation**			
Adequate	94	130	0.765
Inadequate	30	45	
Caecal intubation	104 (83.9%)	151 (86.3%)	0.561

Transient abdominal cramps were common complication in both groups of patients. The clinical features of dehydration were recorded in 2 patients on PEG-ELS from the cathartic effect resulting in one procedure cancellation.

## Discussion

**Figure 2:**
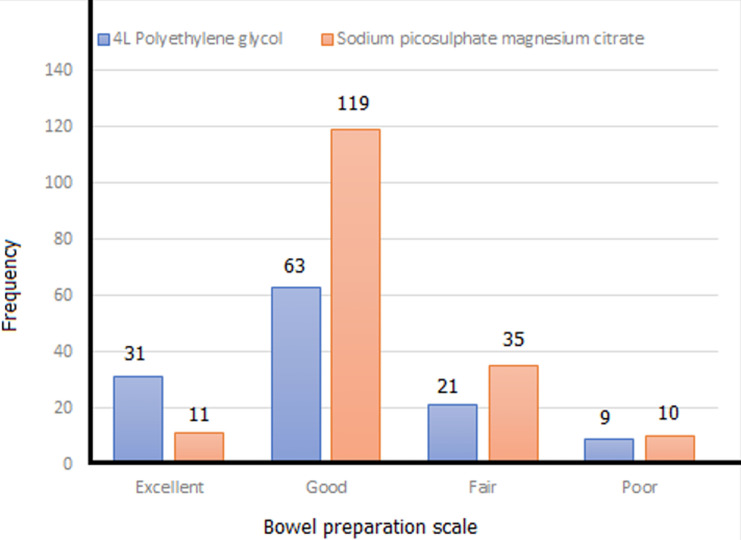
aronchick bowel preparation scale in study patients

Bowel cleansing is a cornerstone for optimal colonoscopy outcome. This comparative colonoscopy study was conducted on out-patients in an ambulatory care endoscopy facility of Niger Delta Nigeria. The sociodemographic of the patient groups was similar as the proportion of patients aged ≥ 50 years was 66.1% in the PEG group and 70.9% in the SPMC group; male gender predominance noted. A higher frequency of excellent grade of bowel preparation was observed with PEG -25.8% (32/124), in comparison to 6.3% (11/175) for SPMC group (Figure 2). However, an overall assessment of adequacy of bowel preparation (excellent/good grades) sodium picosulfate was observed to have a similar efficacy to polyethylene glycol (p=0.592) in this African population. This efficacy profile corroborates high level evidence from comparative study reports of PEG versus SPMC conducted in out-patients from Europe, Asia and North America [19-21].

The use of both oral and written instructions for bowel preparation, as opposed to written instructions only, has been shown to be an independent predictor of adequate level of cleansing [22]. There was an inferred high literacy and ability to comprehend instructions amongst patients as only 6 (2.0%) patients were documented to have formal education limited to primary school level. In the study protocol, a written informative literature with instructions was given to literate patients. Non-adherence to preparation instruction is a predictor of poor level of bowel preparation [23]. An attempt to obviate this with follow-up phone calls from the study centre was made. There was full compliance in all but one case of the SPMC group and 2 in the PEG group.

Polyp and adenoma detection rates are widely used as surrogate for the quality of colonoscopy with influencing factors including quality of bowel preparation, withdrawal time and experience of the endoscopist [24]. A withdrawal time of a minimum of 6 minutes was observed during colonoscopies. There was a statistical difference noted in both polyp and adenoma detection rates in favour of the SPMC group. This is most probably endoscopist-related as polyethylene glycol was primarily used in the early years of endoscopist´s practice. From the centre´s audit the adjusted caecal intubation rate of the endoscopist rose from 75.5% in 2014 to 93.5% by the year 2020 [25]. In normal daily colonoscopy practice completion (caecal intubation rate) is approximately 83% in symptomatic patients and up to 95% for elective colonoscopy [26]. The experience of endoscopist is a strong determinant of polyp and adenoma detection rates. It is a known fact that colorectal cancer incidence and mortality can be reduced by removal of premalignant polyps.

In comparing the safety profile of agents, magnesium citrate is not safe in patients with renal failure as magnesium is solely excreted from the kidney [27]. Polyethylene glycol (PEG); however, is relatively safe for bowel preparation in patients who cannot tolerate a significant fluid load (renal failure, congestive heart failure, or advanced liver disease with ascites) [28]. Albeit no case of renal failure or congestive heart failure was recorded. It is reported that bisacodyl can cause abdominal cramping and ischemic colitis, especially at a dose of ≥10 mg [29, 30]. Transient abdominal cramps were the most common complications noted in both study groups; however, no case of ischemic colitis was recorded. It is unclear if the abdominal cramps were due to bowel distension resulting from air and luminal fluid which usually improve with passage of flatus and absorption. There were two cases of dehydration in patients that received PEG and necessitated cancellation of one procedure.

The practice of colonoscopy introduced in recent years into our environment is not highly utilized and marred by non-ready availability of local supplies of preferred bowel cleansing agents. Other available bowel cleansing alternatives were often used due to non-availability of preferred cleansing agents hence the long duration encountered for the acquisition of data for this comparative study. Endoscopy equipment repairs and maintenance were challenges encountered even with the procurement of new endoscopes. Major repairs involve shipment abroad with a long turn-around-time and logistic challenges of importation clearance with relevant agencies.

## Conclusion

There is a similar outcome in efficacy using either split volume PEG-ELS and SPMC prior to colonoscopy in Nigerian patients. Polyp and adenoma detection rates are highly dependent on the expertise of the endoscopist.

### What is known about this topic

- Colonoscopy is a sensitive tool for screening and diagnosis of colorectal cancer with the added option of polyp removal and resection of early-stage cancer;
- Inadequate bowel cleansing can result in missed lesions, shortened surveillance times, significant impediment in progression of colonoscope or incomplete study;
- There are systematic reviews and meta-analyses comparing the efficacy of polyethylene glycol and sodium picosulfate magnesium citrate for bowel preparation during colonoscopy however, lacking in studies from typical high fibre diet consuming African patients.

### What this study adds

- There is similar efficacy profile using either split volume 4L PEG or SPMC prior to colonoscopy in this Nigerian population;
- A mandatory 2-day dietary restriction to low residue/fluid diet instead of a 1-day restriction, in a typical high-fibre diet population, probably contributed to effective outcome of bowel preparation using these preparation agents;
- Bleeding per rectum is the most common indication for colonoscopy in the study population.
